# Zeaxanthin Inhibits Hypoxia-Induced VEGF Secretion by RPE Cells through Decreased Protein Levels of Hypoxia-Inducible Factors-1*α*

**DOI:** 10.1155/2015/687386

**Published:** 2015-01-20

**Authors:** Richard Rosen, Tommaso Vagaggini, Yueqin Chen, Dan-Ning Hu

**Affiliations:** ^1^Department of Ophthalmology, The New York Eye and Ear Infirmary of Mount Sinai Health System, 310 E. 14th Street, New York, NY 10003, USA; ^2^Icahn School of Medicine at Mount Sinai, New York, NY 10029, USA; ^3^Tissue Culture Center, The New York Eye and Ear Infirmary of Mount Sinai Health System, 310 E. 14th Street, New York, NY 10003, USA; ^4^Department of Pathology, The New York Eye and Ear Infirmary of Mount Sinai Health System, 310 E. 14th Street, New York, NY 10003, USA

## Abstract

Hypoxia is the most important stimulus leading to upregulation of VEGF in the retina and this is caused by accumulation of hypoxia-inducible factors-1*α* (HIF-1*α*) protein. The effects of zeaxanthin, a natural phytochemical, on the VEGF and HIF-1*α* expression in the primary culture of human retinal pigment epithelial (RPE) cells were studied. An in vitro RPE cell hypoxia model was established by placing cells under 1% oxygen pressure or by adding cobalt chloride (CoCl_2_) to the culture medium. RPE cells and conditioned media were collected from cultures treated with and without zeaxanthin under normoxic and hypoxic conditions. VEGF and HIF-1*α* protein and RNA levels were measured by ELISA kits and RT-PCR, respectively. Hypoxia caused a significant increase of VEGF expression and accumulation of HIF-1*α* in RPE cells. Zeaxanthin at 50–150 *μ*M significantly inhibited the expression of VEGF and accumulation of HIF-1*α* protein caused by hypoxia but did not affect expression of VEGF and HIF-1*α* under normoxic conditions. This is the first report on the effect of zeaxanthin on VEGF and HIF-1*α* levels in cultured RPE cells and suggests that zeaxanthin may have potential value in the prevention and treatment of various retinal diseases associated with vascular leakage and neovascularization.

## 1. Introduction

Vascular endothelial growth factor (VEGF) is a growth factor that stimulates the proliferation and migration of vascular endothelial cells and increases vascular permeability [1, 2]. VEGF is the most powerful angiogenesis promoter and plays a significant role in the pathogenesis of ocular neovascularization diseases, such as diabetic retinopathy and exudative type of age-related macular degeneration (AMD) [3–9].

The most important pathophysiological stimulus leading to high levels of VEGF expression in the retina is hypoxia [3, 4, 7, 9]. Hypoxia causes the increase of VEGF by the accumulation of a transcription factor, hypoxia-inducible factor-1*α* (HIF-1*α*), which promotes the production and secretion of VEGF by various cells including retinal pigment epithelial (RPE) cells [10–26]. Hypoxia is closely related to the development of neovascularization in the eye. Any drug that inhibits this process may have a therapeutic effect on various retinal diseases related to neovascularization.

Zeaxanthin, a natural phytochemical, is a carotenoid pigment of the xanthophyll subclass with a chemical formula C_40_H_56_O_2_. Zeaxanthin is present in various tissues and, in particular, is highly concentrated in the central retina (macula) of the eye [27–32]. Various observational and interventional studies have indicated that zeaxanthin might help reduce the risk of age-related macular degeneration (AMD) [33–41]. In vitro studies have demonstrated that zeaxanthin protected RPE cells and various retinal neurons against oxidative stress [42–46]. Zeaxanthin is an antioxidant and also works as a filter protecting the macula from the blue light [42–47]. Recent studies have shown that in addition to traditional mechanisms, zeaxanthin can influence the viability and function of cells through various signal pathways or transcription factors [46, 48].

It has been reported that zeaxanthin decreased the upregulation of VEGF in the retina in diabetic rats and in apoliprotein deficient mice [49, 50]. Lutein, a carotenoid with similar structure and function as zeaxanthin, decreases high VEGF expression of human RPE cells or mouse macrophages following tumor necrosis factor-*α* and lipopolysaccharide stimulations, respectively [51]. To the best of our knowledge, the effects of zeaxanthin on VEGF expression in cultured RPE cells have not been previously reported.

The purposes of the present study were to investigate the effects of zeaxanthin on the expression and secretion of VEGF by RPE cells under normoxic and hypoxic conditions and to explore the mechanism of action by measurement of HIF-1*α* levels in RPE cells under normoxia and hypoxia, with and without zeaxanthin.

## 2. Materials and Methods

### 2.1. Cell Culture

A culture of primary human RPE cells was isolated from a donor eye (56 years old) and cultured as previously described [52, 53]. Cells were cultured in Dulbecco's modified Eagle's medium (DMEM, GIBCO, Carlsbad, CA) supplemented with 10% fetal bovine serum (FBS, GIBCO). Cells were incubated in a humidified 5% CO_2_ atmosphere at 37°C. After reaching confluence, cells were detached by trypsin-EDTA solution (GIBCO), diluted at 1 : 3-1 : 4, plated for subculture, and passaged routinely at a dilution of 1 : 3-1 : 4 every 5–7 d.

Phase-contrast microscopy revealed pigmentation of RPE cells during the primary culture and the first and second subcultures. Cells displayed characteristic epithelial morphology throughout the culture period. The purity of the cell lines was demonstrated by immunocytochemical methods as previously reported. RPE cells display positive staining of cytokeratin, whereas fibroblasts and melanocytes do not [54].

### 2.2. Effect of Hypoxia on Secretion of VEGF by RPE Cells

RPE cells were seeded into 24-well plates at a density of 1 × 10^5^ cells per well. After 24 h, the culture medium was withdrawn. The cultures were washed with PBS twice and fresh culture medium was added. In the hypoxia experiment, cells were incubated in a sealed chamber at 37°C for 24 h in a controlled environment of 1% O_2_ in the presence of 5% CO_2_ and 94% N_2_ by using a PROOX 100 System (BioSherix, Redfield, NT). Cells cultured under standard conditions (21% O_2_, 5% CO_2_, and 74% N_2_) served as normoxia control cultures. Conditioned medium was collected 24 hours later and centrifuged at 800 ×g for 5 min, and the supernatants were transferred to vials and stored at −70°C until analysis. All experiments were performed in triplicate.

### 2.3. Effect of Chemical Hypoxia on Secretion of VEGF by RPE Cells

RPE cells were seeded into 24-well plates as described above. Culture medium was replaced 24 h after seeding and cobalt chloride (CoCl_2_) (Sigma, St. Louis, MO), an iron analogue, was added into the medium to mimic hypoxic conditions. The CoCl_2_ concentrations used in the literatures had a wide variation [55–57]. Therefore, the dose effects of CoCl_2_ were tested over a wide range of CoCl_2_ (at concentrations of 50, 100, 150, and 200 *μ*M). Cells cultured without CoCl_2_ served as normal controls. Conditioned medium was collected 24 h later, centrifuged, and stored as described above. All experiments were performed in triplicate.

### 2.4. Effect of Zeaxanthin on Secretion of VEGF by RPE Cells under Normoxia

RPE cells were seeded into 24-well plates at a density of 1 × 10^5^ cells per well. After 24 h, the culture medium was withdrawn. The cultures were washed with PBS twice and fresh culture medium was added. Zeaxanthin (ZeaVision LLC, Chesterfield, MO) 6.82 mg was dissolved in 200 *μ*L DMSO to make a stock solution of 60 mM. Tested cells were treated by different concentrations of zeaxanthin. The cells in the control group were cultured in medium containing the same levels of DMSO as in the zeaxanthin solution. A separate investigation of the effects of the highest DMSO levels (1 : 400) used in this experiment did not show significant differences in the cell viability between the cells with and without DMSO (data not shown). Conditioned medium was collected 24 h later, centrifuged, and stored as described above. All experiments were performed in triplicate.

### 2.5. Effect of Zeaxanthin on the Secretion of VEGF by RPE Cells under Hypoxia

RPE cells were seeded into 24-well plates and the culture medium was replaced 24 h later as described above. Zeaxanthin was added to the medium at different concentrations. One hour later, cells were incubated in a controlled environment of 1% O_2_ as described above. Cells cultured under hypoxia without zeaxanthin were used as positive controls. Cells cultured under normoxic conditions were used as negative controls. Conditioned medium was collected 24 h later, centrifuged, and stored as described above. All experiments were performed in triplicate.

### 2.6. Effect of Zeaxanthin on the Secretion of VEGF by RPE Cells under Chemical Hypoxia

RPE cells were seeded into 24-well plates and the culture medium was replaced 24 h later as described above. CoCl_2_ was added to mimic hypoxic conditions. Cells cultured with CoCl_2_ but without zeaxanthin were used as positive controls. Cells cultured without CoCl_2_ were used as negative controls. Conditioned medium was collected 24 h later, centrifuged, and stored as described above. All experiments were performed in triplicate.

### 2.7. Effects of Hypoxia and Zeaxanthin on Cell Viability of RPE Cells

RPE cells were seeded into 96-well plates at a density of 5 × 10^3^ cells per well. Cells were incubated under normoxic or hypoxic conditions (1% O_2_) or with added CoCl_2_ at various concentrations. In the study of effects of zeaxanthin on the cell viability, zeaxanthin at 1, 50, 100, and 150 *μ*M was added into the culture medium under normoxic condition. After 24 h, MTT solution (1 mg/mL, 50 *μ*L) was added. After 4 h incubation, the medium and MTT were aspirated and 100 *μ*L of DMSO was added. Optical density of the plates was determined with a microplate reader (Multiskan MCC/340, Fisher Scientific, Pittsburgh, PA, USA) at 540 nm [58]. The optical density in control (normoxic) cells was taken as 100% viability. All tests were performed in three independent experiments.

### 2.8. Measurement of VEGF Levels

The amount of VEGF protein in the conditioned media was determined using the human VEGF Quantikine ELISA kits (R&D Systems, Minneapolis, MN, USA) according to the manufacturer's instructions. Optical density was read using a microplate reader at 450 nm and corrected with 540 nm. The amount of VEGF (pg/mL) was calculated from a standard curve. The sensitivity of the VEGF kits was 5.0 pg/mL.

### 2.9. RNA Isolation and RT-PCR

RPE cells were seeded into 6-well plates and the culture medium was replaced 24 h later as described above. Zeaxanthin was added to the medium at 150 *μ*M. One hour later, cells were incubated in a controlled environment of 1% O_2_ as described above. Cells cultured with 1% O_2_ but without zeaxanthin were used as positive controls. Cells cultured under normoxic conditions were used as negative controls. After 24 h, the culture medium was withdrawn, the cultures were washed with cold PBS, and cells were harvested. After microcentrifuging at 800 ×g for 5 min at 4°C, cell pellets were collected for mRNA extraction. Total RNA was isolated with the RNeasy mini kit (QIAGEN, Valencia, CA), according to the manufacturer's instructions. The SuperScript first-strand synthesis system for RT-PCR kit (Invitrogen, Camarillo, CA) was used to perform cDNA synthesis. The PCR primers for glyceraldehyde-3-phosphate dehydrogenase (GAPDH) were TGAACTGAAAGCTCTCCACC and CTGATGTACCAGTTGGGGAA.* VEGF* primers were AGGGCAGAACATCACGAAGT and ACGGTCTCGATTGGATGGCA.* HIF-1* primers were GAACGTCGAAAAGAAAAGTCTCG and CCTTATCAAGATGCGAACTCACA. All primers were obtained from Invitrogen. The first-strand cDNAs were synthesized from 1.0 *μ*g of total RNA at 50°C for 50 min. PCR amplification was conducted in a GeneAmp PCR system 9700 (Applied Biosystems, Foster City, CA) using the following parameters: first denaturation at 94°C for 5 min followed by 35 cycles of reactions of denaturation at 94°C for 30 s, annealing at 58°C for 45 s, and extension at 72°C for 45 s and last extension for 5 min at 72°C. After amplification, samples were run on a 1% agarose gel (Invitrogen) in TBE (0.01 M Tris-borate) and 0.001 M EDTA (Invitrogen) containing 2.0 *μ*g/mL ethidium bromide (Invitrogen). Bands were visualized and photographed on a UV transilluminator (ChemiDoc XRS System, Bio-Rad, Hercules, CA, USA).

### 2.10. Effect of Hypoxia and Zeaxanthin on HIF-1*α* Protein Levels in RPE Cells

RPE cells were seeded into 6 cm culture dishes at a density of 2 × 10^6^ cells per well. After 24 h, the culture medium was replaced as described above. One hour later, cells were incubated in a controlled environment of 1% O_2_ or with added CoCl_2_ at various concentrations. Cells cultured under normoxic condition and without CoCl_2_ were served as normal controls. After 24 h, cells were collected as described above and treated with cell lysis buffer (SIGMA) and centrifuged at 2000 ×g for 5 min and the supernatant was collected. Protein levels were measured with BCA Protein Assay Kit (Thermo Scientific, Rockfield, IL). In zeaxanthin studies, zeaxanthin at different concentrations was added to the culture medium; 1 h later, cells were incubated under 1% O_2_ or added with CoCl_2_ at 150 *μ*M. Cells cultured under normoxic condition and without CoCl_2_ served as negative controls. Cells cultured under O_2_ or with CoCl_2_ but without zeaxanthin served as positive controls.

### 2.11. Measurement of HIF-1*α* Levels in the Cell Extracts

The amount of HIF-1*α* protein in the cell extracts was determined using the Human/Mouse Intracellular HIF-1*α* DuoSet IC ELISA kits (R&D Systems, Minneapolis, MN, USA) according to the manufacturer's instructions. Optical density was read by using a microplate reader at 450 nm and corrected with 540 nm. The amounts of HIF-1*α* (pg/mL) were calculated from a standard curve and expressed as pg/mg protein.

### 2.12. Statistical Analysis

Statistical significances of the difference of means throughout this study were calculated using the ANOVA one-way test for comparing data from more than two groups and Student's *t*-test for comparing data between two groups. A difference at *P* < 0.05 was considered to be statistically significant.

## 3. Results

### 3.1. Effects of Hypoxia and Zeaxanthin on Cell Viability of RPE Cells

**Figure 1:**
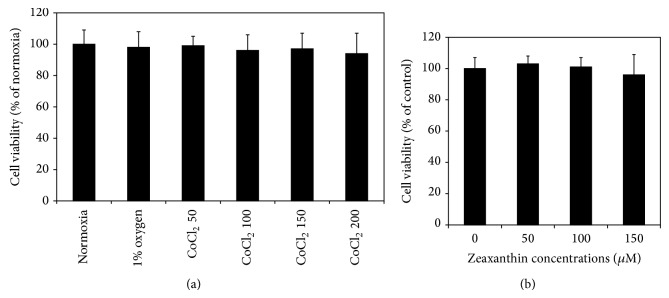
Effects of hypoxia and zeaxanthin on cell viability of retinal pigment epithelial (RPE) cells. RPE cells were seeded into 96-well plates at a density of 5 × 10^3^ cells per well. Cells were incubated under normoxic or hypoxic conditions (1% O_2_) or cultured with CoCl_2_ at 50, 100, 150, and 200*μ*M (a). Zeaxanthin at 1, 50, 100, and 150*μ*M was added into the culture medium under normoxic condition (b). After 24 h, cell viability was determined by MTT assay (see Section 2). Hypoxia and zeaxanthin at test concentrations did not affect the cell viability (*P* > 0.05). Data are expressed as the percentage of optical readings in normoxia (mean ± SD, *n* = 3).

RPE cells cultured at 1% O_2_ or with CoCl_2_ at 50–200 *μ*M did not significantly affect the cell viability (*P* > 0.05). Zeaxanthin at 50–150 *μ*M also did not significantly affect RPE cell viability (*P* > 0.05) (Figure 1).

### 3.2. Secretion of VEGF by RPE Cells under Normoxia and Hypoxia

**Figure 2:**
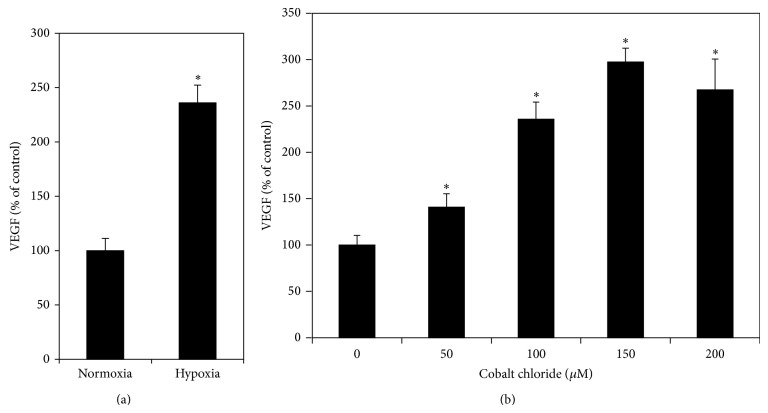
Effects of hypoxia on secretion of VEGF by RPE cells. RPE cells were seeded into 24-well plates at a density of 1 × 10^5^ cells per well. After 24 h, cells were incubated in a sealed chamber in a controlled environment of 1% O_2_ (a) or cultured with CoCl_2_ at 50, 100, 150, and 200*μ*M concentrations (b). Cells cultured under normal oxygen pressure (21% O_2_, 5% CO_2_, and 74% N_2_) were served as the control. Conditioned medium was collected 24 h later; VEGF levels were measured by VEGF ELISA kits and expressed as percentages of the control (mean ± SD, *n* = 3). Hypoxia significantly increased VEGF secretion by RPE cells. ^*^ *P* < 0.05, compared with the controls.

RPE cells had a relatively high constitutive secretion of VEGF. VEGF levels in conditioned culture medium of RPE cells cultured under normoxia were 451 ± 50.8 pg/mL (mean ± SD). Cells cultured under hypoxia (1% O_2_) significantly increased the VEGF levels in the culture medium 2.36-fold over cells cultured under normoxia (*P* < 0.05) (Figure 2(a)). CoCl_2_ at concentrations from 50 to 200 *μ*M caused a dose-dependent increase of VEGF levels (all CoCl_2_ treated cultures compared to the control, *P* < 0.05; 50 *μ*M compared to 100 *μ*M and 100 *μ*M compared to 150 *μ*M, *P* < 0.05; 150 *μ*M compared with 200 *μ*M, *P* > 0.05) (Figure 2(b)). CoCl_2_ stimulated effects on secretion of VEGF reached the peak at 150 *μ*M, which induced a 2.97-fold increase in secretion of VEGF as compared with cells cultured under normoxia. Therefore, 150 *μ*M CoCl_2_ was selected as the concentration of CoCl_2_ used in the following experiments.

### 3.3. Effect of Zeaxanthin on Secretion of VEGF by RPE Cells under Normoxia and Hypoxia

**Figure 3:**
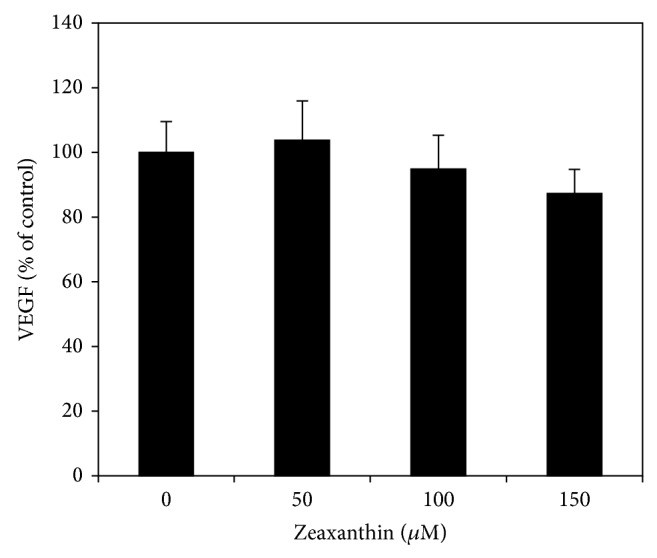
Effect of zeaxanthin on secretion of VEGF by RPE cells under normoxia. RPE cells were seeded into 24-well plates at a density of 1 × 10^5^ cells per well. After 24 h, zeaxanthin at 50, 100, and 150*μ*M was added. Conditioned medium was collected 24 h later; VEGF levels were measured by VEGF ELISA kits and expressed as percentages of the control (mean ± SD, *n* = 3). Zeaxanthin did not significantly affect the secretion of VEGF in cells cultured under normoxia (*P* > 0.05).

Under normoxic condition, zeaxanthin at 50–100 *μ*M did not influence the secretion of VEGF by RPE cells (*P* > 0.05) (Figure 3). Cells cultured with zeaxanthin at 150 *μ*M showed a slight decrease of VEGF secretion as compared with cells not cultured with zeaxanthin; however, this difference was not statistically significant (*P* > 0.05).

**Figure 4:**
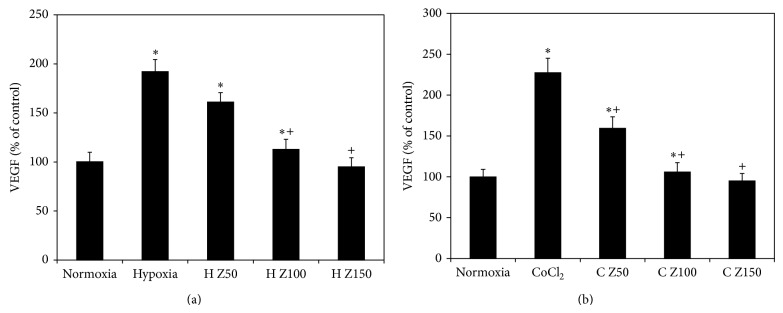
Effects of zeaxanthin on secretion of VEGF by RPE cells under hypoxia. RPE cells were seeded into 24-well plates at a density of 1 × 10^5^ cells per well. After 24 h, zeaxanthin was added at 50, 100, and 150*μ*M concentrations. One hour later, cells were incubated under 1% O_2_ (a) or cultured with CoCl_2_ at 150*μ*M (b). Cells cultured under normoxia and without zeaxanthin were served as negative control. Cells cultured under hypoxia and without zeaxanthin were served as positive control. Conditioned medium was collected 24 hours later; VEGF levels were measured by VEGF ELISA kits and expressed as percentages of the negative control (mean ± SD, *n* = 3). Zeaxanthin significantly inhibited hypoxia-induced secretion of VEGF. ^*^ *P* < 0.05, compared with the negative controls. ^+^ *P* < 0.05, compared with the positive controls.

Under hypoxic condition (1% O_2_), zeaxanthin at 50–150 *μ*M dose-dependently decreased the secretion of VEGF as compared with cells cultured under hypoxia but without zeaxanthin (compared to hypoxia without zeaxanthin, *P* > 0.05 at 50 *μ*M and *P* < 0.05 at 100–150 *μ*M) (50 *μ*M compared to 100 *μ*M, *P* < 0.05; 100 *μ*M compared to 150 *μ*M, *P* > 0.05) (Figure 4(a)). Secretion of VEGF in cells cultured with zeaxanthin at 150 *μ*M decreased to levels near those of cells cultured under normoxia (*P* > 0.05), indicating that zeaxanthin at 150 *μ*M completely blocked hypoxia-induced secretion of VEGF by RPE cells.

In the chemical hypoxic condition (150 *μ*M CoCl_2_), zeaxanthin at 50–150 *μ*M also dose-dependently decreased the secretion of VEGF as compared to cells cultured under chemical hypoxia but without zeaxanthin (*P* < 0.05 at all concentrations of zeaxanthin) (50 *μ*M compared to 100 *μ*M and 100 *μ*M compared to 150 *μ*M, *P* < 0.05) (Figure 4(b)). Zeaxanthin at the highest tested concentrations also completely blocked CoCl_2_-induced secretion of VEGF by RPE cells (*P* > 0.05, as compared with cells cultured without zeaxanthin).

### 3.4. Effects of Hypoxia and Zeaxanthin on Expression of* VEGF* mRNA

**Figure 5:**
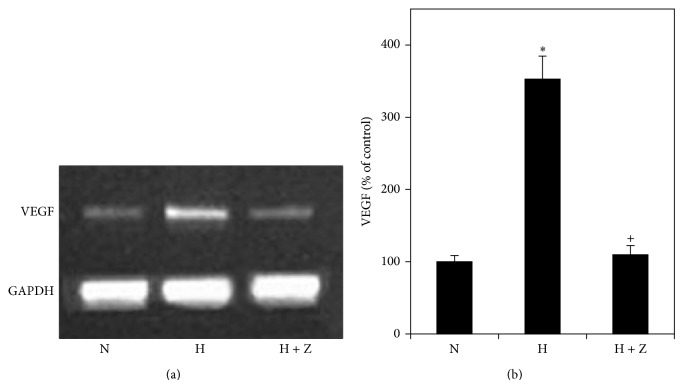
Effects of hypoxia and zeaxanthin on* VEGF* mRNA expression of RPE cells. (a) Representative RT-PCR profiles from three experiments. Cells were cultured under hypoxia (1% oxygen) with (H + Z) and without zeaxanthin (H). Cells cultured under normal oxygen condition were used as the control (N). Cells were collected 24 hours later, mRNA was extracted, and RT-PCR analysis was performed as described in Section 2.* GAPDH* was used as an internal loading control. (b) Quantitative analysis showed that the expression of* VEGF* mRNA (mean ± SD, *n* = 3) by cells exposed to hypoxia (1% oxygen) was significantly increased (H) and zeaxanthin significantly inhibited hypoxia-induced expression of* VEGF* (H + Z). ^*^ *P* < 0.05, compared with the controls.

Expression of* VEGF* mRNA in RPE cells increased significantly in cells cultured with 1% O_2_ for 24 h (*P* < 0.05) (Figure 5). Adding of zeaxanthin at 150 *μ*M significantly decreased the expression of* VEGF* mRNA (*P* < 0.05) (Figure 5), indicating that zeaxanthin blocked hypoxia-induced expression of* VEGF* mRNA.

### 3.5. Effects of Hypoxia on Intracellular HIF-1*α* Protein Levels in RPE Cells

**Figure 6:**
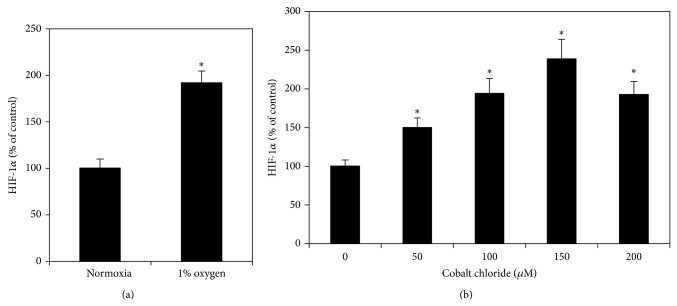
Effect of hypoxia on HIF-1*α* protein levels in RPE cells. RPE cells were seeded into 6 cm culture dishes at a density of 2 × 10^6^ cells per well. After 24 h, cells were incubated in a controlled environment of 1% O_2_ (a) or added with CoCl_2_ (b) at 50, 100, 150, and 200*μ*M. Cells cultured under normoxic condition and without CoCl_2_ served as normal controls. Cells were collected 24 h later; HIF-1*α* protein levels in cell extracts were determined by using the Intracellular HIF-1*α* ELISA kits and expressed as percentages of the control (mean ± SD, *n* = 3). Hypoxia significantly increased HIF-1*α* protein levels in RPE cells. ^*^ *P* < 0.05, compared with the controls.

RPE cells cultured under hypoxia (1% O_2_) produced significantly increased levels of HIF-1*α* protein to 1.92-fold of cells cultured under normoxia (*P* < 0.05) (Figure 6(a)). CoCl_2_ at all concentrations from 50 to 200 *μ*M caused a dose-dependent increase of HIF-1*α* protein levels in RPE cells (*P* < 0.05) (all the CoCl_2_ treated group at different dosages compared to the next group, *P* < 0.05) (Figure 6(b)). CoCl_2_ stimulated effects on HIF-1*α* levels reached the peak at 150 *μ*M, which induced a 2.39-fold increase in HIF-1*α* levels as compared with cells cultured without CoCl_2_. Therefore, 150 *μ*M CoCl_2_ was selected as the concentration of CoCl_2_ used in the following experiments.

### 3.6. Effects of Zeaxanthin on Intracellular HIF-1*α* Protein Levels in RPE Cells

**Figure 7:**
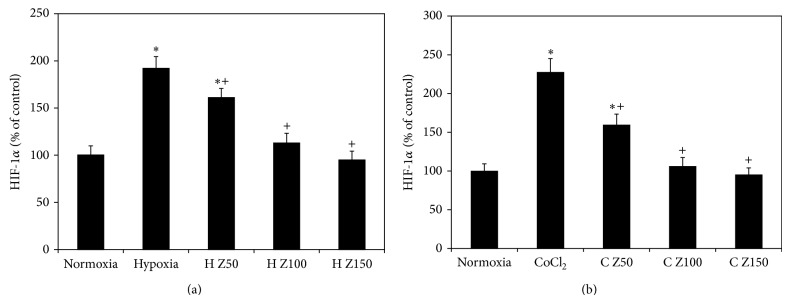
Effect of zeaxanthin on HIF-1*α* protein levels in RPE cells under hypoxia. RPE cells were seeded into 6 cm culture dishes at a density of 2 × 10^6^ cells per well. After 24 h, zeaxanthin was added at 50, 100, and 150*μ*M concentrations. One hour later, cells were incubated under 1% O_2_ (a) or cultured with CoCl_2_ at 150*μ*M (b). Cells cultured under normoxic condition and without zeaxanthin served as negative controls. Cells cultured under hypoxia and without zeaxanthin served as positive control. Cells were collected 24 h later; HIF-1*α* protein levels in cell extracts were determined by using the Intracellular HIF-1*α* ELISA kits and expressed as percentages of negative control (mean ± SD, *n* = 3). Zeaxanthin significantly inhibited hypoxia-induced accumulation of HIF-1*α* protein. ^*^ *P* < 0.05, compared with the negative controls. ^+^ *P* < 0.05, compared with the positive controls.

In cells cultured with 1% O_2_, zeaxanthin at 50–150 *μ*M caused a dose-dependent decrease of HIF-1*α* levels as compared with cells cultured under 1% O_2_ but without zeaxanthin (*P* < 0.05 at all concentrations of zeaxanthin) (50 *μ*M compared to 100 *μ*M, *P* < 0.05; 100 *μ*M compared to 150 *μ*M, *P* > 0.05) (Figure 7(a)). HIF-1*α* levels in cells cultured with 1% O_2_ and zeaxanthin at 100–150 *μ*M showed no difference from cells cultured under normoxia (*P* > 0.05), indicating that zeaxanthin at 100–150 *μ*M could completely block hypoxia-induced intracellular accumulation of HIF-1*α* protein.

In cells cultured with CoCl_2_, zeaxanthin at 50–150 *μ*M also caused a dose-dependent decrease of HIF-1*α* (*P* < 0.05 at all concentrations of zeaxanthin compared with cells cultured with CoCl_2_ but no zeaxanthin) (50 *μ*M compared to 100 *μ*M, *P* < 0.05; 100 *μ*M compared to 150 *μ*M, *P* > 0.05) (Figure 7(b)). Zeaxanthin at 100–150 *μ*M completely blocked CoCl_2_-induced accumulation of HIF-1*α* protein (*P* > 0.05, as compared with cells cultured without CoCl_2_).

### 3.7. Effects of Hypoxia and Zeaxanthin on Expression of* HIF-1α* mRNA

**Figure 8:**
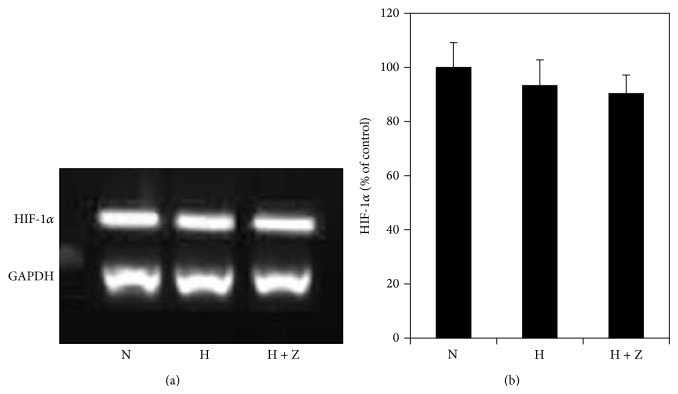
Effects of hypoxia and zeaxanthin on* HIF-1α* mRNA expression of RPE cells. (a) Representative RT-PCR profiles from three experiments. Cells were cultured under hypoxia (1% oxygen) with (H + Z) and without zeaxanthin (H). Cells cultured under normal oxygen condition were used as the control (N). Cells were collected 24 hours later, mRNA was extracted, and RT-PCR analysis was performed as described in Section 2.* GAPDH* was used as an internal loading control. (b) Quantitative analysis showed that the* expression of HIF-1α* mRNA (mean ± SD, *n* = 3) was not affected by being exposed to hypoxia or zeaxanthin (*P* > 0.05).

Cells cultured under 1% O_2_ with and without zeaxanthin did not show any significant effect on the expression of* HIF-1α* mRNA (*P* > 0.05) (Figure 8). This indicates that both hypoxia and zeaxanthin have no effect on the expression of* HIF-1α*. Their effects on the HIF-1*α* protein levels are mainly via the stabilization and accumulation process of HIF-1*α* protein. This is consistent with previous reports [17, 18].

## 4. Discussion

In vivo and in vitro studies have indicated that the most important pathophysiological stimulus leading to high expression of VEGF in the retina is hypoxia [3, 4, 7, 9]. Hypoxia causes the increase of VEGF by RPE cells mainly through the stabilization of HIF-1*α* protein [17–22].

HIF-1 is a transcription factor for cellular and tissue adaptation to low oxygen tension and is the most important factor promoting angiogenesis via upregulation of VEGF under hypoxia [23–26]. HIF-1 is a heterodimeric factor consisting of an inducible oxygen-sensitive alpha subunit (HIF-1*α*) and a constitutive oxygen-insensitive beta subunit (HIF-1*β*/ARNT). The expression of HIF-1*β* is not affected by changes of oxygen pressure. Cells continuously synthesize and degrade HIF-1*α* protein. The protein level of HIF-1*α* is tightly regulated by cellular oxygen concentration [23–26]. Under normoxic conditions, HIF-*α* subunits have a very short half-life; the proline residues in the oxygen-dependent degradation domain of HIF-1*α* are hydroxylated by prolyl hydroxylase. Subsequently, the hydroxylated HIF-1*α* is recognized by the Von Hippel-Lindau tumor suppressor protein, leading to ubiquitination and degradation of HIF-1*α* and thereby abolishing HIF-1*α* protein accumulation. Under hypoxia, the hydroxylation of HIF-1*α* is impaired, which enhances stabilization and accumulation of HIF-1*α* protein. Upon HIF-1*α* protein accumulation, it translocates to the nucleus, stimulates expression of* VEGF* gene, and leads to angiogenesis [23–26].

In this in vitro study, the hypoxic condition was induced by changing the oxygen pressure in the incubator or by adding CoCl_2_ [10–22, 55–57]. Under normoxia, HIF-1*α* is degraded by the hydroxylases. The ferrous ion bound at the active sites is essential for the activity of hydroxylases. Cobalt displaces the single free ferrous at the active site and thus deactivates the hydroxylases [59]. Therefore, cobalt is able to stabilize HIF-1*α* protein and it has been used widely in experiments for producing chemical hypoxia [55–57, 60].

It has been reported that hypoxia-induced expression of* VEGF* mRNA and secretion of VEGF by RPE cells could be produced by culturing the cells under low oxygen environment (usually 1% oxygen) [10–16] or by adding CoCl_2_ (100–200 *μ*M) into the culture medium [55–57]. Hypoxia induces accumulation of HIF-1*α* protein levels in RPE cells by culturing the cells in hypoxia [17–22] or by using CoCl_2_ [55, 60].

In the present study, hypoxia significantly induced expression of* VEGF* and accumulation of HIF-1*α* protein in cultured RPE cells. These results are consistent with the previous reports [10–22, 55–57].

Zeaxanthin is a carotenoid pigment and belongs to the xanthophyll subclass with a chemical formula C_40_H_56_O_2_. It is found at high levels in various foods (e.g., egg yolk, corn, and many vegetables and fruits). Zeaxanthin is present in the tissues and is highly concentrated in the retina, especially in the macula [27–32].

Epidemiologic studies suggest that insufficient dietary lutein and zeaxanthin intake and lower levels of lutein and zeaxanthin in the retina or serum may be associated with increased risk for AMD [33–36]. Several but not all supplementation studies or clinical trials have shown that supplementation of zeaxanthin with other antioxidants may have a favorable effect on the prevention and treatment of AMD [37–40]. Recently, a meta-analysis study on 6 longitudinal cohort studies indicated that dietary intake of lutein and zeaxanthin was significantly related to a reduction of risk for late AMD but not for early AMD [41].

One in vitro study showed that zeaxanthin significantly reduced lipofuscin formation in photoreceptor outer segment-fed RPE cells [45]. Zeaxanthin reduces photooxidative damage and decreases upregulation of expression of IL-8 (a proinflammation and angiogenic chemokine) in RPE cells caused by A2E and blue light irradiation [43]. Zeaxanthin protects cultured photoreceptors against oxidative stress caused by H_2_O_2_ or paraquat [46].

In an experimental animal study, zeaxanthin combined with other antioxidants increased retinal antioxidants activity and slowed down the photoreceptor degeneration in a retinitis pigmentosa model (rd1 mouse) [61].

Zeaxanthin protects the retina against oxidative stresses by two mechanisms: it acts as an antioxidant or as a blue light filter [47]. In addition to these two traditional mechanisms, recent studies found that zeaxanthin and its closely related molecule (lutein) may affect the growth, viability, and functions of various cell types via different signal pathways, transcription factors, growth factors, and cytokines [46, 48].

Experimental animal studies showed that zeaxanthin could decrease the upregulation of* VEGF* in the retinal-choroid tissues in apolipoprotein deficient mice [50] and prevent diabetes-induced increase of retinal VEGF in diabetic rats [49]. Lutein has been reported to decrease high* VEGF* expression following tumor necrosis factor-*α* stimulation of human RPE cells and inhibits lipopolysaccharide-induced* VEGF* expression in mouse macrophages [51]. A preliminary report suggested that adding oral zeaxanthin treatment (20 mg/day) to an aggressive treatment regimen (bevacizumab, steroid, and PDT therapy) for choroidal neovascularization (CNV) improved therapeutic efficiency and reduced the number of PDT therapies. The progression to CNV in the fellow eye in zeaxanthin treated patients was reduced to 50% of patients without zeaxanthin [62]. However, the effects of zeaxanthin on* VEGF* expression in cultured RPE cells have not been previously reported.

In the present study, zeaxanthin did not cause a significant change in constitutive VEGF secretion by cultured human RPE cells. Zeaxanthin significantly reduced hypoxia-induced expression of* VEGF* mRNA and secretion of VEGF by RPE cells in a dose-dependent manner. Zeaxanthin at a higher concentration could completely block hypoxia-induced expression and secretion of VEGF by RPE cells. Zeaxanthin also inhibited hypoxia-induced intracellular accumulation of HIF-1*α*, which is the main transcription factor involved in hypoxia-induced expression of VEGF.

## 5. Conclusions

In the present study, zeaxanthin blocked hypoxia-induced VEGF secretion in cultured RPE cells but not constitutive secretion of VEGF. Therefore, the possible detrimental effects caused by complete local blocking of VEGF might be avoided through the use of high-dose zeaxanthin supplementation [63–66]. Additionally, zeaxanthin, by inhibition of hypoxia-induced accumulation of HIF-*α*1 protein, may have a broader effect on the control of angiogenesis caused by factors other than VEGF [26, 67]. Zeaxanthin taken orally could be used as an adjunct to intravitreal anti-VEGF therapy enabling a decrease in the frequency of injections with reduced risk of local side effects [68–70]. Therefore, zeaxanthin might be a promising agent to be explored for the prevention and treatment for a variety of retinal diseases associated with revascularization.
